# Factors Related to the Visiting Nurse Staff's Intention to Continue Working

**DOI:** 10.1002/nop2.70656

**Published:** 2026-07-07

**Authors:** Tatsuhito Kamimoto, Sumie Ikezaki, Mami Onishi

**Affiliations:** ^1^ Graduate School of Nursing Chiba University Chiba Japan; ^2^ Fundamental Nursing National Defense Medical College Saitama Japan

**Keywords:** cross‐sectional study, intention to continue working, visiting nurse

## Abstract

**Aim:**

This study aimed to examine the factors related to the visiting nurse staff's intention to continue working and to obtain nursing management implications for their continued employment.

**Design:**

A quantitative cross‐sectional study.

**Methods:**

A nationwide self‐administered survey was conducted among visiting nurses in Japan (July–October 2021) using a hybrid response mode (online and paper). Questionnaires were targeted for distribution to 2500 nurses: 2400 via visiting nurse stations selected through stratified random sampling across all prefectures and an additional ~100 via supplementary convenience/purposive recruitment to improve response rates. Intention to continue working was dichotomised (agree/strongly agree vs. disagree/strongly disagree), and multivariable logistic regression was used to identify factors independently associated with intention to continue working.

**Results:**

A total of 276 responses were received (response rate 11.0%). After excluding managers, 182 staff visiting nurses were analysed; 130 (71.4%) reported intention to continue working. Higher perceived managerial leadership was strongly associated with intention to continue working (OR = 11.70, 95% CI = 3.46–39.50), as was being married (OR = 2.58, 95% CI = 1.07–6.21).

**Conclusions:**

Managerial leadership may be a key, modifiable organizational factor associated with visiting nurses' intention to continue working. Strengthening managerial leadership may contribute to the retention of visiting nurses.

**Patient or Public Contribution:**

No patient or public involvement occurred in the design or conduct of this study.

## 1 Introduction

With a large proportion of older people worldwide, Japan is entering a superaged society (Desa 2019), and the demand for medical care is expected to increase. Changes in Japan's health care system, such as shorter hospitalization and a shift in the place of care to the home, will increase the demand for home health care and necessary human resources. In Japan, the ‘Home‐Visit Nursing Action Plan 2025’ formulated in 2014 by the Home‐Visit Nursing Promotion Collaborative Conference established by the Japan Nursing Association, the Japan Visiting Nurse Foundation, and the National Association of Home‐Visit Nursing Services established a goal of increasing the number of visiting nurses to approximately 150,000 (Japan Visiting Nurse Foundation 2017). However, there were approximately 70,000 employed visiting nurses in 2022 (Ministry of Health, Labour and Welfare 2024). This shortage of nurses is occurring worldwide (Canadian Federation of Nurses Unions 2012; American Association of Colleges of Nursing 2014; International Council of Nurses 2020). According to the International Council of Nurses (2006), this shortage includes poor working conditions and high turnover caused by burnout (Chan et al. 2013). The turnover rate for visiting nurses in Japan is approximately 15% (Japanese Nursing Association 2011), which is close to the 14% turnover rate for visiting nurses in the U.S. (Ellenbecker et al. 2008). The turnover rate for hospital nurses in Japan is approximately 11% (Japanese Nursing Association 2022), with a higher turnover rate for visiting nurses. Several studies have highlighted the importance of retaining experienced visiting nurses to ensure patient safety and quality of care (Tang and Hudson 2019; Markle‐Reid et al. 2006). Thus, it is critical to consider measures to support the continued employment of visiting nurses and prevent their turnover to enhance home health care.

One's intention to continue working affects actual job retention (Ellenbecker et al. 2008). Other studies have examined factors related to the job retention of visiting nurses, including age, years of home care nursing experience and the presence of dependents (Anthony and Milone‐Nuzzo 2005). Influential factors, such as job satisfaction (Ellenbecker 2004), the length of conversations with managers and coworkers (Noguchi‐Watanabe et al. 2020), and ‘time pressure’ from having to travel to the subsequent destination (Cao and Naruse 2019), are related to the intention to continue working or to leave the job. Beyond these visiting‐nurse–specific findings, broader evidence across nursing settings suggests that retention‐related outcomes are shaped by modifiable organizational factors, particularly leadership and the practice environment.

Recent evidence across nursing settings suggests that nurses' retention and intention to stay are shaped by modifiable organizational factors, particularly leadership and the practice environment. Systematic reviews have consistently reported associations between supportive leadership, adequate staffing and resources, positive practice environments, and lower turnover intention or improved retention outcomes (Pressley and Garside 2023; de Vries et al. 2023). In addition, studies informed by the Job Demands–Resources (JD–R) perspective indicate that strengthening job resources (e.g., managerial support and organizational resources) may buffer the negative impact of job demands such as workload and stress and may support nurses' willingness to remain (Kaihlanen et al. 2023). Although much of the evidence on practice environments has been generated in hospital contexts (Figueiredo et al. 2025), these findings underscore the potential importance of organizational resources in community‐based services, including visiting nursing, where autonomy and dispersed work conditions may amplify the role of managerial leadership and organizational support.

Taken together, these perspectives highlight the need to examine how organizational resources (work environment) and job demands (e.g., workload and on‐call duties) jointly relate to visiting nurses' intention to continue working, particularly using multivariable approaches. Despite reports on the factors influencing visiting nurses' intention to continue working, the workload, such as the number of visits or night shift standby duties that influence the intention, has not been verified. Factors influencing the intention to continue working need to be examined in a multivariate analysis, as it is considered that various factors may be influencing the intention to continue working. Previous research indicates that a positive work environment enhances job retention by improving nursing care quality, patient outcomes and reducing staff turnover (Rodríguez‐García et al. 2020). For home health care nurses in small organizations, factors such as the presence of management and the work environment significantly influence their intention to continue working. Delving into specific elements of the work environment could yield valuable insights. A significant challenge in visiting nursing research is the dominance of univariate studies that examine the impact of different factors on nurses' intentions to continue working, with a lack of multivariate analyses. Consequently, it's crucial to incorporate various factors, such as the work environment, into multivariate analysis. This approach will provide a more comprehensive understanding of the factors influencing Japanese visiting nurses' decisions to intention to continue working.

We aimed to explore the factors influencing the intention to continue working among Japanese visiting nurses. By clarifying the relevant factors, we intended to obtain suggestions for specific support measures for visiting nurses to continue their employment and to help optimize their workload in managing their organizations.

## 2 The Study

### 2.1 Aim of Study

The overall aim of this study was to examine factors associated with visiting nurses' intention to continue working and to derive implications for nursing management to support retention.

### 2.2 Research Question and Objectives

Research question: Which individual and organizational factors, including the work environment measured by the Work Environment Scale for Visiting Nurses (WESVN), are independently associated with visiting nurses' intention to continue working in Japan?

Objectives: (1) To examine the associations between visiting nurses' intention to continue working and individual and organizational characteristics, including the work environment measured by the WESVN. (2) To identify independent factors associated with intention to continue working using multivariable logistic regression. (3) To derive nursing management implications to support the continued employment of visiting nurse staff based on the identified factors.

## 3 Methods

### 3.1 Study Design

A quantitative cross‐sectional design was adopted because the study aimed to estimate the prevalence of intention to continue working and to examine associations between multiple individual and organizational factors and a binary outcome using multivariable modelling. This approach aligns with recommendations for quantitative appraisal in nursing research (Cathala and Moorley 2018).

### 3.2 Participants and Sampling

The target population comprised visiting nurses working clinically in visiting nurse stations across Japan. We used a two‐stage recruitment approach.

Stage 1: Stratified random sampling of stations. Visiting nurse stations were selected using the ‘Nursing Facility Search’ information system provided by the Ministry of Health, Labour and Welfare. The sampling frame comprised all stations listed in the database at the time of sampling (2021). To minimize geographic bias, stations were stratified by prefecture and randomly selected within each prefecture using a random number table. We then asked each selected station to distribute the questionnaire to up to three eligible visiting nurses (staff nurses providing direct clinical care). This procedure targeted the distribution of questionnaires to 2400 nurses from 800 stations (three nurses per station).

Stage 2: To improve response rates, we conducted supplementary recruitment by inviting stations and nurses willing to participate from across Japan and asked participating stations to distribute questionnaires to approximately 100 nurses from ~15 stations. This supplementary recruitment can be considered convenience/purposive in nature and was used pragmatically to enhance participation, consistent with guidance on sample selection in nursing research (Shorten and Moorley 2014). We acknowledge that this supplementary approach may limit generalisability; therefore, it was treated as complementary to the primary stratified random sampling.

Eligibility criteria: Participants were eligible if they were employed as visiting nurse staff providing direct clinical care. Responses from those who identified their role as ‘manager’ were excluded from analysis.

### 3.3 Data Collection and Survey Period

Data collection was performed with an anonymous self‐report questionnaire survey, using both the internet survey method with Google Forms and a mail survey method, allowing the respondents to select the response method. In the case of the mail survey method, we requested the respondents to fill in their answers directly on the enclosed questionnaire and return them in a sealed envelope. The survey period ranged from July 01, 2021, to October 31, 2021.

### 3.4 Survey Items

The dependent variable was the intention to continue working, and the independent variables were individual and organizational characteristics.

#### 3.4.1 Individual Characteristics

The following 17 items were used as individual characteristics: age, sex, employment status, marital status, education, ownership, years of experience at the current workplace, average number of nursing visits per week, average number of on‐call nights per week and annual income.

#### 3.4.2 Organizational Characteristics

The work environment in visiting nursing organizations was measured using the Work Environment Scale for Visiting Nurses (WESVN) (Kamimoto and Onishi 2023), which has been demonstrated to have reliability and validity. The WESVN is a four‐point scale that consists of 35 items and measures five factors: resource flexibility and adequacy (12 items), managerial leadership (9 items), education and career support for nursing quality (8 items), inter‐professional work (3 items), and support system for nursing practice (3 items). The responses ranged from ‘strongly disagree’ (1 point) to ‘strongly agree’ (4 points). The total scores of the WESVN range from 35 to 140.

#### 3.4.3 Intention to Continue Working

The rating scale was a 4‐point scale ranging from ‘strongly disagree’ (1 point) to ‘strongly agree’ (4 points). For inferential analyses, responses were dichotomised into ‘intention to continue working’ (agree/strongly agree) versus ‘no intention to continue working’ (disagree/strongly disagree).

### 3.5 Analysis

Respondents who answered ‘strongly agree’ or ‘agree’ in the ‘intention to continue working’ category were defined as the ‘intention to continue working’ group. Descriptive analyses were conducted using means and standard deviations (SDs), frequencies and percentages. In bivariate analysis, we used independent sample *t*‐tests and Chi‐square tests. Multiple logistic regression analysis was conducted using the forced‐entry method for the variables significant in the bivariate analyses. A multivariable logistic regression model was used because the dependent variable (intention to continue working) was dichotomised (yes/no). This approach enabled estimation of adjusted odds ratios for intention to continue working. To address intercorrelations among WESVN subscales, we fitted separate models with each subscale entered individually. To prepare for entering the score of the WESVN sub‐scale, we performed a Z‐transformation on the score to calculate the size of the factor. To investigate the working environment factors precisely, we compared the model discrimination when each sub‐scale of WESVN was entered because the WESVN sub‐scales had a high correlation coefficient among each subscale. The model discrimination was compared with the AUC (ROC curve). All Statistical analysis was performed using SPSS version 27 (IBM, Armonk, NY, USA) with a *p*‐value < 0.05 as significant.

### 3.6 Theoretical/Conceptual Framework

This study was guided by a job resources perspective consistent with the Job Demands–Resources (JD–R) model, which posits that organizational resources (e.g., supportive leadership, adequate resources and career support) can foster favourable work‐related outcomes and reduce withdrawal cognitions, including intention to leave (or conversely, intention to stay/continue working). According to the JD–R model (Demerouti et al. 2001; Schaufeli and Bakker 2004), job resources initiate a motivational process that is associated with favourable outcomes and reduced turnover intentions. In visiting nurse settings, multiple aspects of the practice environment may operate as job resources, particularly given the dispersed and autonomous nature of community‐based work. Accordingly, we conceptualized the work environment assessed by the Work Environment Scale for Visiting Nurses (WESVN)—including resource flexibility and adequacy, managerial leadership, education and career support, inter‐professional work and support systems for nursing practice—as organizational resources that may be associated with visiting nurses' intention to continue working. We examined how these workplace resources relate to intention to continue working while adjusting for individual characteristics in multivariable analyses.

### 3.7 Rigour (Validity, Reliability and Dependability)

To enhance methodological rigour, we used established measures where available and reported sampling, procedures and analyses transparently to support reproducibility (dependability). The work environment was assessed using the Work Environment Scale for Visiting Nurses (WESVN), which was developed and psychometrically evaluated by the authors using data from the same survey dataset; evidence of its validity and reliability has been published (Kamimoto and Onishi 2023). The WESVN was administered in Japanese as originally developed. In the present analytic sample, internal consistency was high (Cronbach's α = 0.95 for the total scale; subscale α = 0.75–0.91).

A formal pilot study of the overall questionnaire was not conducted because the instrument primarily comprised previously validated measures, and the key organizational measure (WESVN) had already undergone psychometric evaluation within the same survey dataset.

To support data quality, the survey was administered anonymously and respondents were offered two response modes (online and mail) to reduce burden. For the mail survey, participants returned the questionnaire in a sealed envelope to maintain confidentiality. Returned datasets were checked for completeness and consistency prior to analysis.

Analytic rigour was enhanced by using multivariable logistic regression to estimate adjusted associations while accounting for multiple individual and organizational characteristics. Because WESVN subscales were moderately to highly correlated, we fitted separate models with each WESVN subscale entered individually to reduce multicollinearity; model discrimination was evaluated using the area under the receiver operating characteristic curve (AUC).

Response rate: We report the total number of questionnaires targeted for distribution (2400 via the primary stratified random sampling route and 100 via supplementary recruitment), the number of responses received, and the analytic sample in the Results section to enable assessment of potential non‐response bias. We acknowledge that selection and non‐response bias may affect generalisability and address these limitations in the Discussion. The present study focused on examining associations with intention to continue working rather than re‐estimating the measurement model.

### 3.8 Ethical Considerations

This study was approved by the Research Ethics Committee of the Graduate School of Health Care and Nursing, Juntendo University (Approval No. 2021‐50; approved on August 26, 2021). All participants were informed regarding their right to refuse participation and provided their written informed consent prior to answering the questionnaire.

## 4 Results

### 4.1 Response Rate

The questionnaire was targeted for distribution to 2500 visiting nurses (2400 via the primary stratified random sampling route and 100 via supplementary recruitment). A total of 276 responses were received, yielding an overall response rate of 11.0%. Of these, 222 responses (80.4%) were obtained through the primary stratified random sampling route and 54 responses (19.6%) through supplementary recruitment. Because this study focused on visiting nurse staff, 66 respondents who reported their job title as ‘manager’ were excluded. In addition, 28 respondents with missing data on 20% or more of the key study variables were excluded. As a result, the final analytic sample comprised 182 visiting nurses.

### 4.2 Descriptive Statistics

#### 4.2.1 Individual Characteristics

The mean age was 41.2 ± 9.2 years; there were 163 (89.6%) women. In total, 148 (81.3%) visiting nurses were employed full‐time, and 81 (44.5%) were employed at for‐profit corporations; 65 (35.7%) visiting nurses had been working at their current workplace for 1 year to < 3 years and 59 (32.4%) averaged 16 to 20 home nursing visits per week. The mean number of on‐call nights per week was 5.1 ± 3.0 times (Table 1).

**TABLE 1:** A comparison of individual and organizational characteristics with the intention to continue working among Visiting Nurse.

	*N* = 182			
	*n* (%)/mean ± SD	Yes (*n* = 130)	No (*n* = 51)	*p*
Individual characteristics				
Age	41.2 ± 9.2	41.6 ± 9.2	40.0 ± 9.0	0.312^a^
Sex				
Male	18 (9.9)	13 (10.0)	5 (9.8)	0.968^b^
Female	163 (89.6)	117 (90.0)	46 (90.2)	
Employment status				
Full‐time	148 (81.3)	106 (81.5)	42 (82.4)	0.898^b^
Part‐time	33 (18.1)	24 (18.5)	9 (17.6)	
Marital status				
Married	132 (72.5)	103 (79.2)	29 (56.9)	0.002^b^
Single	49 (26.9)	27 (20.8)	22 (43.1)	
Education				
Associate nursing school	5 (2.7)	5 (3.8)	0 (0)	0.393^b^
Department of advanced health care	5 (2.7)	4 (3.1)	1 (0.8)	
College of higher education	11 (6.0)	8 (6.2)	3 (2.3)	
Nursing school	109 (59.9)	79 (60.8)	30 (23.1)	
Junior college	15 (8.2)	12 (9.2)	3 (2.3)	
University	34 (18.7)	20 (15.4)	14 (10.8)	
Graduate school master's program	2 (1.1)	2 (1.5)	0 (0)	
Graduate school doctoral program	0 (0.0)	0 (0)	0 (0)	
Ownership				
Local government	4 (2.2)	4 (3.1)	0 (0.0)	0.365^b^
Social welfare corporations	14 (7.7)	11 (8.5)	3 (5.9)	
Medical corporation	45 (24.7)	34 (26.2)	11 (21.6)	
Associations and foundations	13 (7.1)	11 (8.5)	2 (3.9)	
For‐profit corporations (companies)	81 (44.5)	52 (40.0)	29 (56.9)	
Non‐profit organization (NPO)	3 (1.6)	3 (2.3)	0 (0.0)	
Medical association	2 (1.1)	2 (1.5)	0 (0.0)	
Other	19 (10.4)	13 (10.0)	6 (11.8)	
Years of experience at the current workplace				
< 1 years	43 (23.6)	29 (22.3)	14 (27.5)	0.597^b^
1–< 3 years	65 (35.7)	49 (37.7)	16 (31.4)	
3–< 5 years	28 (15.4)	20 (15.4)	8 (15.7)	
5–< 10 years	26 (14.3)	16 (12.3)	10 (19.6)	
10–< 20 years	14 (7.7)	12 (9.2)	2 (3.9)	
20–< 30 years	5 (2.7)	4 (3.1)	1 (2.0)	
The average number of nursing visits per week				
1–5 times	29 (15.9)	24 (18.5)	5 (9.8)	0.514^b^
6–10 times	17 (9.3)	13 (10.0)	4 (7.8)	
11–15 times	19 (10.4)	15 (11.5)	4 (7.8)	
16–20 times	59 (32.4)	41 (31.5)	18 (35.3)	
21–25 times	39 (21.4)	27 (20.8)	12 (23.5)	
26–30 times	8 (4.4)	4 (3.1)	4 (7.8)	
More than 30 times	10 (5.5)	6 (4.6)	4 (7.8)	
The average number of on‐call nights per week				
Times	5.1 ± 3.0	5.3 ± 3.0	4.5 ± 3.0	0.089^a^
Annual income (million yen)				
< 3	45 (24.7)	34 (26.2)	11 (21.6)	0.278^b^
3–< 4	70 (38.5)	49 (37.7)	21 (41.2)	
4–< 5	40 (22.0)	29 (22.3)	11 (21.6)	
5–< 6	22 (12.1)	17 (13.1)	5 (9.8)	
6–< 7	4 (2.2)	1 (0.8)	3 (5.9)	
Intention to continue working				
Strongly agree	57 (31.3)			
Agree	73 (40.1)			
Disagree	36 (19.8)			
Strongly disagree	15 (8.2)			
Organizational characteristics				
WESVN				
Resource flexibility and adequacy	32.3 ± 7.9	34.2 ± 6.8	28.0 ± 8.3	< 0.01^a^
The bottom row shows *Z*‐scores	0.38 ± 0.66			
Managerial leadership	26.8 ± 5.5	28.6 ± 4.4	22.3 ± 5.5	< 0.01^a^
	0.91 ± 0.61			
Education and career support for nursing quality	18.1 ± 5.2	19.1 ± 4.8	15.4 ± 5.5	< 0.01^a^
	0.10 ± 0.65			
Inter‐professional work	8.7 ± 1.7	8.9 ± 1.6	8.4 ± 1.9	0.061^a^
	0.88 ± 0.58			
Support system for nursing practice	8.5 ± 2.1	9.0 ± 1.8	7.2 ± 2.5	< 0.01^a^
	0.82 ± 0.72			
Total score	94.7 ± 18.7	100.4 ± 15.0	82.0 ± 19.8	< 0.01^a^

*Note:* Cases with missing values were excluded from each analysis; therefore, the number of participants varied slightly across variables.

Abbreviation: WESVN, Work Environment Scale for Visiting Nurses (Kamimoto and Onishi 2023).

^a^ *T*‐test.

^b^ Chi‐squared test.

#### 4.2.2 Organizational Characteristics

The Total Score of WESVN was 94.7 ± 18.7, indicating a wide distribution. The mean value per item in WESVN was 2.71 ± 0.53. The mean value per item of each subscale in WESVN was 2.69 ± 0.66 for resource flexibility and adequacy, 2.98 ± 0.61 for managerial leadership, 2.26 ± 0.65 for education and career support for nursing quality, 2.90 ± 0.57 for inter‐professional work, and 2.83 ± 0.70 for support system for nursing practice. The mean value per item of each subscale was the highest for managerial leadership and lowest for education and career support for nursing quality. Overall, subscale mean item scores were in the mid‐to‐high range of the 4‐point scale. The Z‐transformed scores for each subscale of WESVN ranged from 0.10 to 0.91, all positive and nearly 1 (Table 1).

#### 4.2.3 Intention to Continue Working

We used the intention to continue working as a binarized variable. The ‘intention to continue working’ group comprised 130 respondents (71.4%), including 57 (31.3%) who responded ‘strongly agree’ and 73 (40.1%) who responded ‘agree’. The ‘no intention to continue working’ group comprised 51 respondents (28.0%), including 36 (19.8%) who responded ‘strongly disagree’ and 15 (8.2%) who responded ‘disagree’.

### 4.3 Factors Related to the Intention to Continue Working

Marital status was the only individual characteristic that demonstrated a significant difference between the groups (Table 1). Each subscale of WESVN scores was significant differences for resource flexibility and adequacy, managerial leadership, education and career support for nursing quality, and support system for nursing practice.

Logistic regression analysis was conducted using the independent variables that were significant in the univariate analysis as mandatory inputs. Z‐transformed scores of the WESVN were employed in this analysis to ensure that the number of items in each factor group remained unaffected by the analysis. Total scores were excluded since the subscales were utilized in this study. Between the factors significantly related in the bivariate analysis, as presented in Table 2, the results indicated that the odds ratio (OR) for the individual characteristic of marital status was 2.58 (95% confidence interval [CI] 1.07–6.21), and the OR for the organizational characteristic of managerial leadership was 11.70 (95% CI 3.46–39.5).

**TABLE 2:** Factors related to the intention to continue working.

	*N* = 182			
	Adjusted odds ratio	95% confidence interval	*p*	AUC
Marital status (yes = 1, no = 0)	2.58	(1.07–6.21)	0.04*	0.83
Resource flexibility and adequacy	1.10	(0.45–2.68)	0.83	
Managerial leadership	11.70	(3.46–39.5)	< 0.01*	
Education and career support for nursing quality	0.47	(0.15–1.51)	0.21	
Support system for nursing practice	1.55	(0.62–3.91)	0.35	

Abbreviation: AUC, area under the receiver operating characteristic curve.

* *p* < 0.05.

The logistic regression analysis demonstrated that only managerial leadership was a significant WESVN factor. However, the correlations of managerial leadership may be high among each subscale of WESVN; therefore, Pearson's correlation analyses were carried out to confirm the associations among each subscale of WESVN. The results showed that the correlations of managerial leadership were high among each subscale of WESVN (*r* = 0.598–0.712) (Table 3); therefore, we assessed the AUC for the goodness of fit of each statistical model for WESVN subscales and marital status, as these were significant in the bivariate analysis, to confirm the model's reasonable rate and compared with the model fit. The results also showed that similar to the logistic regression analysis results, managerial leadership demonstrated the highest reasonable rate (marital status: OR 2.96, 95% CI 1.29–6.80; managerial leadership: OR 9.56, 95% CI 4.41–20.74, AUC 0.83) (Table 4).

**TABLE 3:** Correlation coefficients for WESVN subscales.

	*N* = 182			
	Resource flexibility and adequacy	Managerial leadership	Education and career support for nursing quality	Support system for nursing practice
Resource flexibility and adequacy	1			
Managerial leadership	0.637*	1		
Education and career support for nursing quality	0.598*	0.712*	1	
Support system for nursing practice	0.608*	0.692*	0.692*	1
Inter‐professional work**	0.277*	0.400*	0.375*	0.357*

Abbreviation: WESVN: Work Environment Scale for Visiting Nurses (Kamimoto and Onishi 2023).

* *p* < 0.01.

** To clarify interscale correlations for all subscales, shown for reference.

**TABLE 4:** Models of entering each subscale of the WESVN as factors.

	*N* = 182			
	Adjusted odds ratio	95% confidence interval	*p*	AUC
Model 1				
Marital status	2.45	(1.14–5.27)	0.02*	0.74
Resource flexibility and adequacy	3.92	(2.09–7.35)	< 0.01*	
Model 2				
Marital status	2.96	(1.29–6.80)	0.01*	0.83
Managerial leadership	9.56	(4.41–20.74)	< 0.01*	
Model 3				
Marital status	2.71	(1.28–5.73)	0.01*	0.72
Education and career support for nursing quality	3.28	(1.80–5.99)	< 0.01*	
Model 4				
Marital status	2.73	(1.27–5.87)	0.01*	0.76
Support system for nursing practice	3.43	(2.03–5.81)	< 0.01*	

Abbreviation: AUC, area under the receiver operating characteristic curve.

* *p* < 0.05.

## 5 Discussion

### 5.1 Relationship Between Individual Characteristics and the Intention to Continue Working

Of 17 individual characteristics, marital status was associated with continued working, and married participants had a higher intention to continue working. The presence of children in the family situation was shown to be associated with continued working (Stewart et al. 2011), which is similar to our results. Work–family conflict influences nurses' turnover intention (Yamaguchi et al. 2016); hence, adjusting the workload to balance work–family life was considered important in the organizational structure.

Previous studies demonstrated that the large amount of work entrusted to nurses generates a sense of burden (Anthony and Milone‐Nuzzo 2005; Mitsumoto et al. 2008), and three or more nights on‐call imposed a mental and physical burden (Kikuchi and Ishii 2016). Therefore, this study explored various workloads that may be related to the intention to continue working, such as the average number of nursing visits per week; however, we obtained no significant results. Therefore, from the viewpoint of job retention, the workload is a mental and physical burden, but it is suggested that workload is not the only factor that directly affects the intention to continue working.

### 5.2 Relationship Between Organizational Characteristics and the Intention to Continue Working

In this study, the bivariate analysis revealed that four out of five WESVN subscales, namely resource flexibility and adequacy, managerial leadership, education and career support for nursing quality, and support system for nursing practice, were significantly associated with the intention to continue working. Model fit rates demonstrated that the OR for these factors ranged from 3.28 to 3.92, indicating a significant influence on the intention to continue working. However, in a logistic regression analysis, only managerial leadership remained significant, with an OR of 11.70 (95% CI 3.46–39.5), indicating that managerial leadership was the most influential factor in the intention to continue working. In other words, it was suggested that the stronger the leadership of the organization's managers towards the visiting nurses, the higher the possibility that the visiting nurse staff would continue to work. This finding is similar to previous studies in that nurse leadership plays a critical role in creating a healthy work environment and influencing nurse retention and patient care quality (Wei et al. 2018).

### 5.3 Multivariate Analysis of the Association Between Leadership and Intention to Continue Working

In this study, we conducted a multivariate analysis of factors associated with intention to continue working that had been reported in previous studies. In the results, we identified marital status and managerial leadership as the key factors that increased the intention to continue working among visiting nurses. Managerial leadership consists of nine items, including ‘managers and supervisors do not blame staff for mistakes or errors, but use them as learning opportunities’, ‘work well done is appreciated and recognized’, and ‘staff opinions are taken into account in solving management problems in the organization’. The implementation of these behaviours by managers may impact the continuity of employment of the visiting nurses. However, the WESVN manager's leadership used in this study is one of the factors that compose the WESVN, which comprehensively measures the work environment. It is difficult to state that this item alone adequately captures managerial leadership among the visiting nurses. Leadership is defined as ‘having a vision, or clear view, of what future state to aim for, then being able to inspire confidence and motivate others to share that view and work together to achieve it’ (Shaw 2007). Certain leadership styles, such as transformational and authentic leadership, affect staff retention, job satisfaction and empowerment (Cummings et al. 2010; Sasaki et al. 2018; Labrague et al. 2020). More significant organizational outcomes can be achieved by identifying the leadership styles that benefit or diminish organizational goals (Specchia et al. 2021); therefore, it is necessary to examine leadership styles and behavioural characteristics effective in the field of visiting nursing in the future. Notably, this study is the first to report the influence of managerial leadership on the intention to continue working among visiting nurses in Japan. These results indicate the importance of managerial leadership in maintaining a stable nursing workforce and improving the quality of care provided by visiting nursing organizations.

Another interesting finding was that the amount of workload is not a key factor that influences the intention to continue working among visiting nurses by simultaneously analysing the factors in a multivariate analysis. This is valuable for the future management of visiting nursing stations. In addition, this study generated novel findings in that we examined organizational characteristics using the WESVN.

### 5.4 Limitations

This study had several limitations. First, we performed a cross‐sectional study and thus could not clarify causal relationships. Second, previous findings were based on surveys conducted in specific regions or prefectures, and this study was conducted on a national scale. Despite these limitations, our results are novel in that we used a scale with reliability and validity to explore the relevant factors that influence the intention of visiting nurses to continue working. Our results will help examine future efforts and support methods in human resource retention and organizational development targeting visiting nurses. Third, the response rate's low figure of around 11%, combined with the use of random sampling and the convenience sampling method, could limit the generalizability of these results. Respondents may have a more positive attitude to their working condition than non‐responses.

## 6 Conclusions

The findings of this study highlight the crucial role of managerial leadership in shaping visiting nurses' intention to remain in their roles. Although marital status was also a significant factor, its influence was less pronounced than leadership. The persistently high turnover rate among visiting nurses poses a severe challenge to Japan's sustainability and quality of home health care services. Therefore, cultivating strong leadership within nursing management is imperative to improve retention rates and ensure the continuity of care.

This study emphasizes the necessity of targeted educational programs to strengthen the leadership capabilities of nursing managers, enabling them to foster supportive and engaging work environments. Additionally, future research should prioritize multivariate analyses to further understand the various factors influencing nurses' job retention, thereby aiding in the development of more comprehensive strategies to address this issue. By investing in leadership development and promoting favourable working conditions, home health care organizations can bolster workforce stability and, consequently, improve the quality of care delivered to patients.

In conclusion, managerial leadership is a significant factor in encouraging the continued employment of visiting nurses. Future research should focus on identifying the leadership styles and behaviours most conducive to visiting nursing and developing educational initiatives to enhance leadership skills, ensuring home health care's long‐term growth and sustainability.

## Author Contributions

Tatsuhito Kamimoto contributed to the conception and design of the study, data collection, data analysis, interpretation of the findings, and drafting of the manuscript. Sumie Ikezaki contributed to the study design, interpretation of the data, and critical revision of the manuscript. Mami Onishi contributed to data collection, interpretation of the data, and critical revision of the manuscript. All authors participated in revising the manuscript, approved the final version, and agree to be accountable for all aspects of the work.

## Funding

The authors have nothing to report.

## Disclosure

AI use statement: No generative AI or AI‐assisted technologies were used in the writing, analysis, or preparation of this manuscript.

## Ethics Statement

This study was approved by the Research Ethics Committee of the Graduate School of Health Care and Nursing, Juntendo University (Approval No. 2021‐50; approved on August 26, 2021). All participants were informed of their right to decline participation, and written informed consent was obtained prior to completion of the questionnaire.

## Consent

Informed consent was obtained from all participants involved in this study.

## Conflicts of Interest

The authors declare no conflicts of interest.

## Data Availability

The data that support the findings of this study are available on request from the corresponding author. The data are not publicly available due to privacy or ethical restrictions.

## References

[nop270656-bib-0001] American Association of Colleges of Nursing . 2014. “Nursing Shortage Fact Sheet.” http://www.aacn.nche.edu/media‐relations/NrsgShortageFS.pdf.

[nop270656-bib-0002] Anthony, A. , and P. Milone‐Nuzzo . 2005. “Factors Attracting and Keeping Nurses in Home Care.” Home Healthcare Nurse 23, no. 6: 372–377. 15956856 10.1097/00004045-200506000-00009

[nop270656-bib-0003] Canadian Federation of Nurses Unions . 2012. “The Nursing Workforce: Canadian Federation of Nurses Union's Backgrounder.” https://nursesunions.ca/factsheet/nursing‐workforce‐backgrounder.

[nop270656-bib-0004] Cao, X. , and T. Naruse . 2019. “Effect of Time Pressure on the Burnout of Home‐Visiting Nurses: The Moderating Role of Relational Coordination With Nursing Managers.” Japan Journal of Nursing Science 16, no. 2: 221–231. 10.1111/jjns.12233. 30203464

[nop270656-bib-0333] Cathala, X. , and C. Moorley . 2018. “How to Appraise Quantitative Research.” Evidence‐Based Nursing 21, no. 4: 99–101. 10.1136/eb-2018-102996. 30228107

[nop270656-bib-0005] Chan, Z. C. Y. , W. S. Tam , M. K. Y. Lung , W. Y. Wong , and C. W. Chau . 2013. “A Systematic Literature Review of Nurse Shortage and the Intention to Leave.” Journal of Nursing Management 21, no. 4: 605–613. 10.1111/j.1365-2834.2012.01437.x. 23406374

[nop270656-bib-0006] Cummings, G. G. , T. MacGregor , M. Davey , et al. 2010. “Leadership Styles and Outcome Patterns for the Nursing Workforce and Work Environment: A Systematic Review.” International Journal of Nursing Studies 47, no. 3: 363–385. 10.1016/j.ijnurstu.2018.04.016. 19781702

[nop270656-bib-0007] de Vries, N. , A. Boone , L. Godderis , et al. 2023. “The Race to Retain Healthcare Workers: A Systematic Review on Factors That Impact Retention of Nurses and Physicians in Hospitals.” Inquiry: The Journal of Health Care Organization, Provision, and Financing 60: 469580231159318. 10.1177/00469580231159318. PMC10014988 36912131

[nop270656-bib-0008] Demerouti, E. , A. B. Bakker , F. Nachreiner , and W. B. Schaufeli . 2001. “The Job Demands‐Resources Model of Burnout.” Journal of Applied Psychology 86, no. 3: 499–512. 10.1037/0021-9010.86.3.499. 11419809

[nop270656-bib-0009] Desa, U. N. 2019. World Population Prospects 2019: Highlights. Vol. 11, 125. United Nations Department for Economic and Social Affairs.

[nop270656-bib-0010] Ellenbecker, C. H. 2004. “A Theoretical Model of Job Retention for Home Health Care Nurses.” Journal of Advanced Nursing 47, no. 3: 303–310. 10.1111/j.1365-2648.2004.03094.x. 15238125

[nop270656-bib-0011] Ellenbecker, C. H. , F. W. Porell , L. Samia , J. J. Byleckie , and M. Milburn . 2008. “Predictors of Home Healthcare Nurse Retention.” Journal of Nursing Scholarship 40, no. 2: 151–160. 10.1111/j.1547-5069.2008.00220.x. 18507570

[nop270656-bib-0012] Figueiredo, A. R. , C. Baixinho , and P. Lucas . 2025. “Nursing Practice Environment Influences on Retention and Turnover Intention: An Umbrella Review.” Collegian 33: 8–17. 10.1016/j.colegn.2025.09.008. PMC11587427 39585126

[nop270656-bib-0013] International Council of Nurses . 2006. “The Global Nursing Shortage: Priority Areas for Intervention.” http://www.icn.ch/images/stories/documents/publications/GNRI/The_Global_Nursing_Shortage‐Priority_Areas_for_Intervention.pdf.

[nop270656-bib-0014] International Council of Nurses . 2020. “The Global Voice of Nursing in the Year of the Nurse and Covid‐19 Pandemic – ICN Annual Report 2020.” https://www.nurse.or.jp/nursing/international/icn/katsudo/pdf/icn_unnual_reports_jpver2020.pdf.

[nop270656-bib-0015] Japan Visiting Nurse Foundation . 2017. “Visiting Nurse Action Plan.” https://www.jvnf.or.jp/2017/actionplan2025.pdf.

[nop270656-bib-0016] Japanese Nursing Association . 2011. “Data on the Growth of Home Care Nursing.” https://www.mhlw.go.jp/stf/shingi/2r9852000001jlr7‐att/2r9852000001jlv6.pdf.

[nop270656-bib-0017] Japanese Nursing Association . 2022. “2021 Home‐Visit Nursing Fact‐Finding Survey Report.” https://www.nurse.or.jp/up_pdf/20220401121744_f.pdf.

[nop270656-bib-0018] Kaihlanen, A. M. , S. Ruotsalainen , V. Väisänen , L. Corneliusson , T. Pesonen , and T. Sinervo . 2023. “Job Demand and Job Resource Factors Explaining Stress and Job Satisfaction Among Home Care Nurses – A Mixed‐Methods Sequential Explanatory Study.” BMC Nursing 22, no. 1: 404. 10.1186/s12912-023-01568-3. 37891583 PMC10612316

[nop270656-bib-0019] Kamimoto, T. , and M. Onishi . 2023. “Houmonkangosi Wo Taisyou to Sita Syokubakankyou Sokutei Syakudo no Kaihatu to Sinraisei･Datousei no Kentou [Development, Reliability, and Validity of a Work Environment Measurement Scale for Visiting Nurses].” Journal of Japan Academy of Nursing Science 43: 143–153. 10.5630/jans.43.143.

[nop270656-bib-0020] Kikuchi, Y. , and N. Ishii . 2016. “Houmonkangosi no Yakan Onko‐Rugyoumu to Hutankan Oyobi Suimin He no Eikyou [Influence on Sleep and Burden on Visiting Nurses Engaged in On‐Call Service During the Night].” Sangyō Eiseigaku Zasshi 58: 271–279. 10.1539/sangyoeisei.16-003-E. 27773887

[nop270656-bib-0021] Labrague, L. J. , C. E. Nwafor , and K. Tsaras . 2020. “Influence of Toxic and Transformational Leadership Practices on Nurses' Job Satisfaction, Job Stress, Absenteeism and Turnover Intention: A Cross‐Sectional Study.” Journal of Nursing Management 28, no. 5: 1104–1113. 10.1111/jonm.13053. 32453901

[nop270656-bib-0022] Markle‐Reid, M. , G. Browne , R. Weir , A. Gafni , J. Roberts , and S. R. Henderson . 2006. “The Effectiveness and Efficiency of Home‐Based Nursing Health Promotion for Older People: A Review of the Literature.” Medical Care Research and Review: MCRR 63, no. 5: 531–569. 10.1177/1077558706290941. 16954307

[nop270656-bib-0023] Ministry of Health, Labour and Welfare . 2024. “Overview of Employed Medical Professionals in the 2022 Report on Public Health Administration and Services.” https://www.mhlw.go.jp/toukei/saikin/hw/eisei/22/dl/gaikyo.pdf.

[nop270656-bib-0024] Mitsumoto, I. , T. Matsushita , and Y. Oura . 2008. “Houmonkangosi no Sigotohutankan Ya Syuugyoukeizokuisi to Gyoumutokusei to no Kanren [Relationship Between the Job Characteristics of Home‐Visiting Nurses and Their Job‐Related Burden and Intention to Continue Working].” Journal of UOEH 30, no. 2: 185–196. 10.7888/juoeh.30.185. 18655547

[nop270656-bib-0025] Noguchi‐Watanabe, M. , N. Yamamoto‐Mitani , Y. Nagami , S. Eltaybani , A. Inagaki , and Y. Taniguchi . 2020. “Homecare Nurses' Length of Conversation and Intention to Remain at the Workplace: A Multilevel Analysis.” Journal of Nursing Management 29: 721–730. 10.1111/jonm.13212. 33179317

[nop270656-bib-0026] Pressley, C. , and J. Garside . 2023. “Safeguarding the Retention of Nurses: A Systematic Review on Determinants of Nurse's Intentions to Stay.” Nursing Open 10, no. 5: 2842–2858. 10.1002/nop2.1588. 36646646 PMC10077373

[nop270656-bib-0027] Rodríguez‐García, M. C. , V. V. Márquez‐Hernández , T. Belmonte‐García , L. Gutiérrez‐Puertas , and G. Granados‐Gámez . 2020. “Original Research: How Magnet Hospital Status Affects Nurses, Patients, and Organizations: A Systematic Review.” AJN, American Journal of Nursing 120, no. 7: 28–38. 10.1097/01.NAJ.0000681648.48249.16. 32541337

[nop270656-bib-0028] Sasaki, M. , Y. Yumoto , N. Morioka , Y. Yonekura , H. Ueno , and Y. Ogata . 2018. “Kangosityou no Ri‐da‐Sippu to Sutahhukangosi no Enpawa‐Mento no Kankei: Bunken Kentou [A Literature Review on the Relationship Between Nurse Managers' Leadership and Staff Nurses' Empowerment].” Journal of the Japan Society for Healthcare Administration 55: 133–142. 10.11303/jsha.55.133.

[nop270656-bib-0029] Schaufeli, W. B. , and A. B. Bakker . 2004. “Job Demands, Job Resources, and Their Relationship With Burnout and Engagement: A Multi‐Sample Study.” Journal of Organizational Behavior 25, no. 3: 293–315. 10.1002/job.248.

[nop270656-bib-0030] Shaw, S. 2007. International Council of Nurses: Nursing Leadership. 1st ed. Blackwell Publishing.

[nop270656-bib-0222] Shorten, A. , and C. Moorley . 2014. “Selecting the Sample.” Evidence‐Based Nursing 17, no. 2: 32–33. 10.1136/eb-2014-101747. 24561511

[nop270656-bib-0031] Specchia, M. L. , M. R. Cozzolino , E. Carini , et al. 2021. “Leadership Styles and Nurses' Job Satisfaction. Results of a Systematic Review.” International Journal of Environmental Research and Public Health 18, no. 4: 1552. 10.3390/ijerph18041552. 33562016 PMC7915070

[nop270656-bib-0032] Stewart, N. J. , C. D'Arcy , J. Kosteniuk , et al. 2011. “Moving on? Predictors of Intent to Leave Among Rural and Remote RNs in Canada.” Journal of Rural Health: Official Journal of the American Rural Health Association and the National Rural Health Care Association 27, no. 1: 103–113. 10.1111/j.1748-0361.2010.00308.x. 21204977

[nop270656-bib-0033] Tang, J. H.‐C. , and P. Hudson . 2019. “Evidence‐Based Practice Guideline: Nurse Retention for Nurse Managers.” Journal of Gerontological Nursing 45, no. 11: 11–19. 10.3928/00989134-20191011-03. 31651984

[nop270656-bib-0034] Wei, H. , K. A. Sewell , G. Woody , and M. A. Rose . 2018. “The State of the Science of Nurse Work Environments in the United States: A Systematic Review.” International Journal of Nursing Sciences 5, no. 3: 287–300. 10.1016/j.ijnss.2018.04.010. 31406839 PMC6626229

[nop270656-bib-0035] Yamaguchi, Y. , T. Inoue , H. Harada , and M. Oike . 2016. “Job Control, Work‐Family Balance and Nurses' Intention to Leave Their Profession and Organization: A Comparative Cross‐Sectional Survey.” International Journal of Nursing Studies 64: 52–62. 10.1016/j.ijnurstu.2016.09.003. 27689509

